# Insights Into the Mechanisms of Brain Endothelial Erythrophagocytosis

**DOI:** 10.3389/fcell.2021.672009

**Published:** 2021-08-02

**Authors:** Jiahong Sun, Prema Vyas, Samar Mann, Annlia Paganini-Hill, Ane C. F. Nunes, Wei Ling Lau, David H. Cribbs, Mark J. Fisher, Rachita K. Sumbria

**Affiliations:** ^1^Department of Biomedical and Pharmaceutical Sciences, School of Pharmacy, Chapman University, Irvine, CA, United States; ^2^Henry E. Riggs School of Applied Life Sciences, Keck Graduate Institute, Claremont, CA, United States; ^3^Pitzer College, Claremont, CA, United States; ^4^Departments of Neurology and Pathology and Laboratory Medicine, University of California, Irvine, Irvine, CA, United States; ^5^Division of Nephrology, Department of Medicine, University of California, Irvine, Irvine, CA, United States; ^6^Institute for Memory Impairments and Neurological Disorders, University of California, Irvine, Irvine, CA, United States

**Keywords:** red blood cell, phosphatidylserine, oxidative stress, brain endothelial erythrophagocytosis, iron deposits, cerebral microhemorrhages

## Abstract

The endothelial cells which form the inner cellular lining of the vasculature can act as non-professional phagocytes to ingest and remove emboli and aged/injured red blood cells (RBCs) from circulation. We previously demonstrated an erythrophagocytic phenotype of the brain endothelium for oxidatively stressed RBCs with subsequent migration of iron-rich RBCs and RBC degradation products across the brain endothelium *in vivo and in vitro*, in the absence of brain endothelium disruption. However, the mechanisms contributing to brain endothelial erythrophagocytosis are not well defined, and herein we elucidate the cellular mechanisms underlying brain endothelial erythrophagocytosis. Murine brain microvascular endothelial cells (bEnd.3 cells) were incubated with tert-butyl hydroperoxide (tBHP, oxidative stressor to induce RBC aging *in vitro*)- or PBS (control)-treated mouse RBCs. tBHP increased the reactive oxygen species (ROS) formation and phosphatidylserine exposure in RBCs, which were associated with robust brain endothelial erythrophagocytosis. TNFα treatment potentiated the brain endothelial erythrophagocytosis of tBHP-RBCs *in vitro*. Brain endothelial erythrophagocytosis was significantly reduced by RBC phosphatidylserine cloaking with annexin-V and with RBC-ROS and phosphatidylserine reduction with vitamin C. Brain endothelial erythrophagocytosis did not alter the bEnd.3 viability, and tBHP-RBCs were localized with early and late endosomes. Brain endothelial erythrophagocytosis increased the bEnd.3 total iron pool, abluminal iron levels without causing brain endothelial monolayer disruption, and ferroportin levels. *In vivo*, intravenous tBHP-RBC injection in aged (17–18 months old) male C57BL/6 mice significantly increased the Prussian blue-positive iron-rich lesion load compared with PBS-RBC-injected mice. In conclusion, RBC phosphatidylserine exposure and ROS are key mediators of brain endothelial erythrophagocytosis, a process which is associated with increased abluminal iron *in vitro*. tBHP-RBCs result in Prussian blue-positive iron-rich lesions *in vivo*. Brain endothelial erythrophagocytosis may provide a new route for RBC/RBC degradation product entry into the brain to produce iron-rich cerebral microhemorrhage-like lesions.

## Introduction

Cerebral microhemorrhages (CMHs) are small focal bleeds in the brain that produce the magnetic resonance imaging signatures of cerebral microbleeds (CMBs) (Greenberg et al., 2009). CMBs increase with normal aging and under pathological conditions and are primarily hemorrhagic in origin (e.g., cerebrovascular disruption resulting in red blood cell (RBC) extravasation) (Martinez-Ramirez et al., 2014; Fisher et al., 2018; Lau et al., 2020). However, since CMBs are clinically identified as small magnetic resonance imaging signal voids resulting from the paramagnetic properties of iron in hemosiderin deposits, sources of CMBs that arise due to an increase in hemosiderin iron *via* alternate mechanisms have been suggested (Fisher, 2014; Janaway et al., 2014).

Under physiological conditions, the adhesion of RBCs to the vascular endothelium is negligible. However, an abnormal RBC-vascular endothelial adhesion has been observed in various pathological conditions (Bonomini et al., 2002; Nakagawa et al., 2011; Wautier and Wautier, 2013; Kucukal et al., 2018). Additionally erythrophagocytosis, a well-described feature of macrophages, by which injured/aged erythrocytes are ingested, digested, and cleared from the blood circulation, has been described in peripheral endothelial cells (Fens et al., 2010, 2012; Lee et al., 2011). A similar phagocytic phenotype has also been reported for the brain endothelium, and studies show that the brain endothelium can engulf and extrude emboli to the abluminal (brain) side *via* angiophagy, *in vivo* and *in vitro* (Grutzendler et al., 2014; van der Wijk et al., 2020). Similarly, our recent *in vitro* and *in vivo* work demonstrated an erythrophagocytic phenotype of the brain endothelium with subsequent passage of iron-rich hemoglobin and RBCs across the endothelial monolayer (Chang R. et al., 2018). The passage of iron-rich RBCs (or RBC degradation products) across an intact brain endothelium following erythrophagocytosis may present an alternate source of iron-rich CMH-like lesions (Fisher, 2014; Janaway et al., 2014; Chang R. et al., 2018).

Phosphatidylserine externalization on RBCs is well-recognized as an important signal that initiates their phagocytic removal from the circulation, and this mechanism is widely studied for macrophage-mediated erythrophagocytosis (de Back et al., 2014). Phosphatidylserine exposure on aged/injured RBCs is also a key trigger for erythrophagocytosis of oxidatively stressed or aged RBCs by peripheral endothelial cells (Fens et al., 2008, 2012). Similarly, our previous study reported a marked externalization of RBC phosphatidylserine by tert-butyl hydroperoxide (tBHP), an oxidative stressor used previously to trigger erythrophagocytosis by peripheral endothelial cells (Fens et al., 2012), and we found robust erythrophagocytosis of tBHP-RBCs by murine brain microvascular endothelial cells (Chang R. et al., 2018). Corroborating our findings, a recent study reported increased erythrophagocytosis of aged RBCs by the brain endothelium, which was associated with increased phosphatidylserine externalization and oxidative stress (Catan et al., 2019). These studies together show an association between RBC phosphatidylserine exposure and oxidative stress, and brain endothelial erythrophagocytosis, but a direct role of RBC phosphatidylserine externalization and oxidative stress in this process has not been reported.

The aim of the current study was to provide mechanistic insights into the cellular pathways involved in brain endothelial erythrophagocytosis. Murine RBCs were treated with tBHP to induce oxidative stress and phosphatidylserine externalization to study the role of these mediators in brain endothelial erythrophagocytosis *in vitro* using murine brain microvascular endothelial cells (bEnd.3). We further examined the impact of brain endothelial erythrophagocytosis on the iron-transport machinery of the bEnd.3 cells and the intracellular trafficking of phosphatidylserine exposing tBHP-RBCs by tracing their movement through early endosomes toward late endosomes, the latter being the presumed site of RBC degradation and release of iron-rich degradation products. Finally, we injected tBHP-RBCs into aged mice to provide histological evidence for increased iron-rich Prussian blue-positive lesion development in mice. Overall, our findings provide important mechanistic insights into the cellular pathways involved in, and the alterations to cellular iron homeostasis associated with, brain endothelial erythrophagocytosis.

## Materials and Methods

Details about the source of the major resources used are in the Supplementary Material.

### Cell Culture

Murine brain microvascular endothelial cells (bEnd.3 cells; American Type Culture Collection, Manassas, VA, United States) were maintained in Dulbecco’s modified Eagle’s medium (American Type Culture Collection, Manassas, VA, United States) supplemented with 10% fetal bovine serum and 100 μg/ml penicillin/streptomycin (Sigma-Aldrich, St. Louis, MO, United States) at standard cell culture conditions (5% CO_2_, 95% air). Cells between passages 22 and 31 were seeded onto 24- and 6-well plates (Corning, New York, NY, United States), 0.2% gelatin-coated glass coverslips, or Transwells with 0.4-μm-pore polyester membrane inserts of a six-well plate (Corning, New York, NY, United States) at a density of 1 × 10^5^ cells/cm^2^, unless otherwise stated.

### RBC Preparation and Treatment for *in vitro* Experiments

RBCs in Alsever’s solution were derived from 2- to 3-month-old male BALB/c mice (BioIVT, New York, NY, United States). RBCs were resuspended in sterile phosphate-buffered saline (PBS, without Ca^2+^ and Mg^2+^; Invitrogen, Waltham, MA, United States) as control, or various concentrations (0.3, 1.0, 3.0 mM) of tBHP (Sigma-Aldrich, St. Louis, MO, United States) at 37°C for 30 min. RBCs at a density of 2 × 10^6^/cm^2^ were co-incubated with bEnd.3 cells with or without the 7.5-μg annexin-V (BioLegend, San Diego, CA, United States) to cloak phosphatidylserine, the antioxidant vitamin C (15–1,500 μM, Sigma-Aldrich, St. Louis, MO, United States), to reduce ROS, or inflammatory cytokine TNFα (10–100 ng/ml, BioLegend, San Diego, CA, United States). The RBC-to-bEnd.3 ratio was ∼5:1 for these experiments.

### ROS Detection

Intracellular ROS were measured by the ROS-reactive fluorescent indicator 2′,7′-dichlorodihydrofluorescein diacetate (H_2_DCFDA, Molecular Probes, Carlsbad, CA, United States). Treated RBCs (2 × 10^6^) were incubated with 10 μM H_2_DCFDA for 30 min at 37°C. The mean fluorescence intensity of DCF was measured at 485 nm/530 nm (excitation/emission) using a fluorescence plate reader (Molecular Devices, LLC, San Jose, CA, United States).

### Phosphatidylserine Exposure Detection and Cloaking, and CD47 Detection

Annexin-V-FITC (BioLegend, San Diego, CA, United States) was used to quantify phosphatidylserine externalization on the RBC surface as per the manufacturer’s instructions. Briefly, 1 × 10^5^ RBCs suspended in annexin-V blocking buffer (BD Biosciences, Franklin Lakes, NJ, United States) were incubated with 5 μg of annexin-V-FITC for 15 min in the dark, followed by immediate analysis of the FITC signal using a BD Accuri C6 Plus flow cytometer (BD Biosciences, San Jose, CA, United States). Ten thousand events were recorded for each sample, and RBCs were quantified using logarithmic gain for light scatter and fluorescence channels with gating and background settings defined through the PBS-RBCs. To determine the correct amount of annexin-V required to block phosphatidylserine exposure, phosphatidylserine-exposing RBCs were first incubated with 7.5 μg annexin V (BioLegend, San Diego, CA, United States) and then labeled with annexin-V-FITC (5 μg), followed by FITC detection using flow cytometry. For CD47 detection, 2.5 × 10^5^ RBCs were incubated with the anti-CD47 antibody (1:200 dilution, Santa Cruz Biotechnology, Dallas, TX, United States) in PBS containing 1% bovine serum albumin (Thermo Fisher Scientific, Waltham, MA, United States) for 45 min at room temperature. After washing with PBS, the anti-CD47 antibody was detected using the Alexa Fluor 647-labeled goat anti-rat IgG (H + L) secondary antibody (1:2,000 dilution, Invitrogen, Waltham, MA, United States) for 30 min in the dark, followed by three PBS washes for flow cytometry detection.

### Hematoxylin and Eosin (H&E) Staining

H&E staining was performed as described previously (Chang R. et al., 2018). bEnd.3 cells were fixed with 4% paraformaldehyde and then stained with hematoxylin (Sigma-Aldrich, St. Louis, MO, United States) and eosin Y (Sigma-Aldrich, St. Louis, MO, United States) and mounted using Permount (Thermo Fisher Scientific, Waltham, MA, United States). Ten continuous view fields from three random areas per coverslip were imaged at × 40 magnification and manually quantified for total RBC number, RBC-to-bEnd.3 ratio (expressed as %),% of bEnd.3 cells positive for RBC, and % of bEnd.3 cells positive for RBC engulfment, as described previously (Chang R. et al., 2018).

### Hemoglobin Measurement

Hemoglobin was measured using 2,7-diaminofluorene (Sigma-Aldrich, St. Louis, MO, United States) as described previously (Chang R. et al., 2018). bEnd.3 cells incubated with RBCs were washed with PBS for total cellular hemoglobin (hemoglobin from attached and engulfed RBCs) or quickly washed with distilled water three times for intracellular hemoglobin (hemoglobin from engulfed RBCs). Cells were lysed with urea lysis buffer (0.2 M Tris–HCl buffer containing 6 M urea, Thermo Fisher Scientific, Waltham, MA, United States) and mixed at a 1:1 ratio with 2,7-diaminofluorene reaction buffer (10 mg 2,7-diaminofluorene in 10 ml urea lysis buffer containing 9% acetic acid and 0.3% hydrogen peroxide). Absorbance was measured at 620 nm using an absorbance plate reader (Molecular Devices, LLC, San Jose, CA, United States). A set of hemoglobin standards (Lee BioSolutions, Maryland Heights, MO, United States) was used to calculate the amount of hemoglobin.

### Cell Viability Assay

bEnd.3 cells (7,500 cells/well) seeded in 96-well plates (Corning, New York, NY, United States) were assessed for cell viability using the Cell Counting Kit-8 assay as per the manufacturer’s instructions (Dojindo Molecular Technologies, Rockville, MD, United States). Absorbance was measured at 450 nm using an absorbance plate reader (Molecular Devices, LLC, San Jose, CA, United States), and cell viability was adjusted to % of bEnd.3 control group (bEnd.3 cells with no RBC co-incubation).

### Immunocytochemistry

After a 48-h incubation with RBCs, bEnd.3 cells were fixed with 2% paraformaldehyde, permeabilized, and blocked with 5% bovine serum albumin (Thermo Fisher Scientific, Waltham, MA, United States) containing 0.1% Triton X-100 (Thermo Fisher Scientific, Waltham, MA, United States). Cells were incubated with Alexa Fluor 647-labeled anti-early endosome antigen 1 (EEA-1) antibody (1:50 in PBS with 0.75% bovine serum albumin and 0.1% Triton X-100; Santa Cruz Biotechnology, Dallas, TX, United States) or rat anti-lysosome-associated membrane protein 1 (LAMP-1) antibody (the original antibody was diluted 1:2 in 50% glycerol followed by 1:5 dilution in PBS with 0.75% bovine serum albumin and 0.1% Triton X-100; Developmental Studies Hybridoma Bank, Iowa City, IA, United States) overnight at 4°C. The anti-LAMP-1 antibody was detected using the Alexa Fluor 647-labeled goat anti-rat IgG (H + L) secondary antibody (1:200, Santa Cruz Biotechnology, Dallas, TX, United States). Coverslips were mounted with UltraCruz Aqueous Mounting Medium with DAPI (Santa Cruz Biotechnology, Dallas, TX, United States) and imaged at × 63 oil immersion objective with a Leica SP5 confocal microscope (Leica, Wetzlar, Germany). Z-stacks were acquired with a z-step size of 0.5 μm. Each experimental group had at least five coverslips, and 10 images per coverslip were analyzed using ImageJ (NIH, Bethesda, MD, United States). Max projection images were used to count nuclei and nuclei positive for EEA-1/LAMP-1 staining as well as the number of RBCs. The number of EEA-1/LAMP-1-positive RBCs was determined by examining each image in a z-stack to find RBCs localized with EEA-1/LAMP-1 in the same plane. We quantified (1) RBC attachment (expressed as % of number of bEnd.3 cells), (2) RBCs localized to the nucleus (expressed as % of total RBC), (3) EEA-1-/LAMP-1-positive RBCs (expressed as % of total RBCs found in the imaging field of view), and (4) RBCs in the nucleus positive for EEA-1-/LAMP-1 (expressed as a % of RBCs in the nucleus).

### Iron Level Measurement

Iron levels in cell samples (total and intracellular iron) and migration of intracellular iron across the bEnd.3 monolayer were measured by Agilent 8900 Triple Quadrupole Inductively Coupled Plasma Mass Spectrometry (ICP-MS; Agilent, Santa Clara, CA, United States). For total cellular and intracellular iron, bEnd.3 cells (9.5 × 10^5^ per well) were seeded in 6-well plates and incubated with 1 × 10^8^ RBCs per well for 48 h. The RBC-to-bEnd.3 ratio was ∼ 25:1 for these experiments. Cells were washed three times with PBS for total cellular iron (iron from attached and engulfed RBCs) or distilled water for intracellular iron (iron from engulfed RBCs). Samples were lysed with 1 ml 4% nitric acid at 80°C for 3 h, after which 250 μl 30% hydrogen peroxide was added and the samples were further digested for 1 h at 90°C.

To measure passage of intracellular iron across the endothelial monolayer, bEnd.3 cells were grown on six-well Transwell inserts (pore size 0.4 μm, Corning New York, NY, United States). The integrity of the bEnd.3 monolayer in Transwell inserts was assessed by measuring the transendothelial electrical resistance using the EVOM2 Epithelial Volt/Ohm Meter and an STX-2 electrode system (World Precision Instruments LLC, Sarasota, FL, United States). After the transendothelial electrical resistance measurements stabilized, 1 × 10^8^ RBCs were added into each insert seeded with the bEnd.3 cells, for 48 h. Apical and basolateral media were collected separately, and 67% nitric acid was added to each sample to result in a final nitric concentration of 4%. Samples were digested for 3 h at 80°C, and 250 μl 30% hydrogen peroxide per 1-ml media sample was added. Samples were further digested at 90°C for 1 h. Standard curves were drawn from calibration blanks with different concentrations (4 nM–4 μM). Iron concentrations from independent samples were calculated based on the calibrated iron standard curve.

### Western Blot

bEnd.3 cells were washed with PBS and lysed with radioimmunoprecipitation assay buffer containing cocktail protease inhibitors (Bio-Rad, Hercules, CA, United States) and boiled with Laemmli buffer and 10% 2-mercaptoethanol (Bio-Rad, Hercules, CA, United States). Blots were probed with anti-transferrin receptor (TfR) antibody (1:1,000 dilution; Thermo Fisher Scientific, Waltham, MA, United States) or anti-ferroportin antibody (1:1,000 dilution; Novus Biologicals, Littleton, CO, United States) overnight at 4°C. Membranes were exposed to the appropriate horseradish peroxidase–conjugated secondary antibodies, followed by chemiluminescence detection (Thermo Fisher Scientific, Waltham, MA, United States). Equal protein loading was controlled by re-probing the membrane with anti–β-actin antibody (1:1,000 dilution; Santa Cruz Biotechnology, Dallas, TX, United States). Chemiluminescence was detected using the UVP ChemiDoc-It TS2 Imager (Upland, CA, United States), and Image J (NIH, Bethesda, MD, United States) was used for Western blot signal quantification.

### *In vivo* Prussian Blue-Positive Iron-Rich Lesion Development

All animal procedures were approved by University of California-Irvine’s Institutional Animal Care and Use Committee and carried out in compliance with University Laboratory Animal Resources regulations. Blood was collected from 17–18-month-old male C57BL/6 mice (National Institute of Aging, Bethesda, MD, United States) and mixed with 3.2% sodium citrate (0.1/0.9 ml of blood). Blood was centrifuged at 500g for 10 min at 4°C, and RBC pellet was washed with PBS three times. Purified RBCs were resuspended in sterile PBS (without Ca^2+^ and Mg^2+^; Invitrogen, Waltham, MA, United States) as control, or 3 mM of tBHP (Sigma-Aldrich, St. Louis, MO, United States) at 37°C for 30 min. After centrifuging at 500g for 5 min at 4°C, the RBC pellet was resuspended in sterile PBS. PBS- or tBHP-RBCs (3 × 10^8^ cells per mouse) were then injected intravenously *via* the retro-orbital route under brief isoflurane anesthesia into different 17–18-month-old male C57BL/6 mice (*n* = 3 per group). Seven days after RBC injection, cardiac perfusion was performed, and brains were harvested and fixed with 4% paraformaldehyde. Hemi-brains were cut into 40-μm coronal sections, and every sixth section was used to examine Prussian blue-positive lesions as described previously (Sumbria et al., 2016). Prussian blue-positive lesions were counted at × 20 magnification by a blinded observer, and digital images were used to calculate lesion size (μm^2^) and positive area (expressed as a percent of total tissue area analyzed) using the NIH ImageJ software 1.62, as described previously (Sumbria et al., 2016).

### Zonula Occludens-1 (ZO-1) Immunostaining

For each mouse (*n* = 3 per group), three coronal sections (600 μm apart and 40 μm thick) prepared as above were used for ZO-1 immunostaining. Briefly, brain sections were incubated in quenching solution (0.2% Triton X-100 (Thermo Fisher Scientific, Waltham, MA, United States) including 20 mM glycine (Sigma-Aldrich, St. Louis, MO, United States) in PBS) for 1 h, followed by blocking with 0.5% bovine serum albumin (Thermo Fisher Scientific, Waltham, MA, United States) containing 0.3% Triton X-100 (Thermo Fisher Scientific, Waltham, MA, United States) for 1 h. Brain sections were incubated with a rabbit ZO-1 polyclonal antibody (1:100 dilution in blocking buffer, Invitrogen, Waltham, MA, United States) overnight at 4°C. The anti-ZO-1 antibody was detected using Alexa Fluor 647-labeled goat anti-rabbit IgG (H + L) secondary antibody (1:400 dilution in blocking buffer, Invitrogen, Waltham, MA, United States). Brain sections were imaged using a × 60 oil immersion objective with the Nikon Ti-Eclipse microscope (Nikon, Melville, NY, United States), and three cortical images were taken per mouse. Z-stacks were acquired, and max projection images were used to quantify the ZO-1-positive area (expressed as a percent of total tissue area analyzed) using ImageJ (NIH, Bethesda, MD, United States).

### Statistical Analysis

The data are shown as means ± SEM. Student’s *t*-test or the Mann–Whitney *U* test was used to compare two independent groups, and one-way analysis of variance (ANOVA) with Holm–Sidak *post hoc* test to compare more than two independent groups. To test the effect of two factors, two-way ANOVA with Holm–Sidak *post hoc* test was used. GraphPad Prism 8.0 (GraphPad Software, Inc., La Jolla, CA, United States) was used for statistical analyses, and a *p* ≤ 0.05 was considered statistically significant.

## Results

### ROS Production and Phosphatidylserine Exposure

**FIGURE 1:**
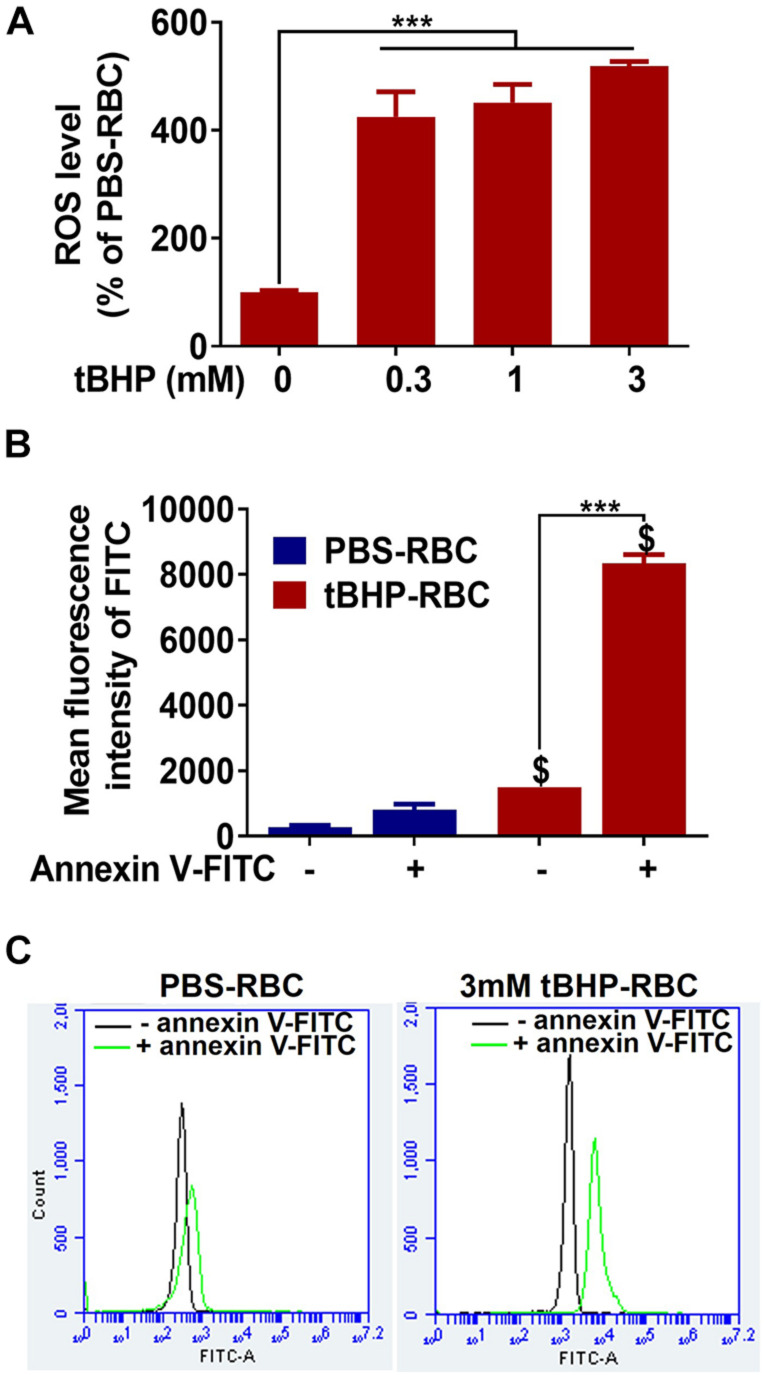
ROS production and phosphatidylserine exposure in PBS- or tBHP-RBCs. ROS level **(A)** and phosphatidylserine exposure **(B,C)** were measured by H_2_DCFDA and annexin-V-FITC, respectively, after a 30-min incubation with PBS or tBHP. Data are presented as mean ± SEM of three independent experiments done in duplicate. One-way **(A)** or two-way **(B)** ANOVA with Holm–Sidak *post hoc* test was used. ***p < 0.001. ^$^*p* < 0.05 compared with the corresponding PBS-RBC group.

As shown in Figure 1A, tBHP (0.3–3 mM) induced a 300–400% increase in ROS levels in RBCs compared with PBS-RBCs. tBHP treatment resulted in a >900% increase in annexin-V-FITC-positive RBCs compared with PBS treatment, indicating significant phosphatidylserine exposure in tBHP-RBCs (Figures 1B,C).

### Temporal Increase in Erythrophagocytosis by bEnd.3 Cells

**FIGURE 2:**
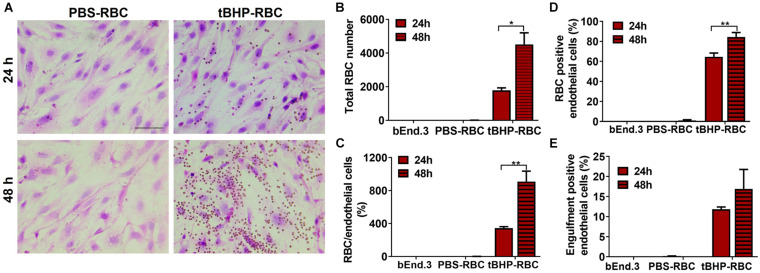
Temporal increase in brain endothelial erythrophagocytosis. Significant time-dependent increase in adhesion **(A–D)** and a trend toward increased engulfment **(E)** of tBHP-RBCs compared with PBS-RBCs using H&E staining and light microscopy. RBCs are seen as small red/brown dots, and bEnd.3 nuclei are stained purple with hematoxylin in A. Data are presented as mean ± SEM of three independent experiments done in duplicates. Two-way ANOVA with Holm–Sidak *post hoc* test was used. *p < 0.05, **p < 0.01. Scale bar = 50 μm.

Our previous study reported a temporal increase in the erythrophagocytosis of tBHP-RBCs (3 mM dose) by the bEnd.3 cells up to 24 h (Chang R. et al., 2018). To further investigate the timeline of erythrophagocytosis of tBHP-RBCs, studies were done following a 24- or 48-h co-incubation of bEnd.3 cells with RBCs. The increased attachment of tBHP-RBCs to the bEnd.3 cells is evident in Figure 2A, where the RBCs appear as small red/brown dots on H&E-stained bEnd.3 cells. Compared with the 24-h timepoint, exposure up to 48 h further increased the number of RBCs attached to the bEnd.3 cells (Figures 2A–C) and the number of endothelial cells positive for tBHP-RBCs (Figure 2D). Although not statistically significant, there was a trend toward an increase in the engulfment of tBHP-RBCs by the bEnd.3 cells at 48 h (Figure 2E). No significant erythrophagocytosis with PBS control was observed at 24 or 48 h (Figures 2A–C). Based on these results, a 48-h co-incubation of RBCs and bEnd.3 was used for subsequent studies. Interestingly, we saw a dose-dependent increase in brain endothelial erythrophagocytosis of tBHP-RBCs with TNFα (10–100 ng/mL) (Supplementary Figure 1).

### Role of Phosphatidylserine Exposure in Brain Endothelial Erythrophagocytosis

**FIGURE 3:**
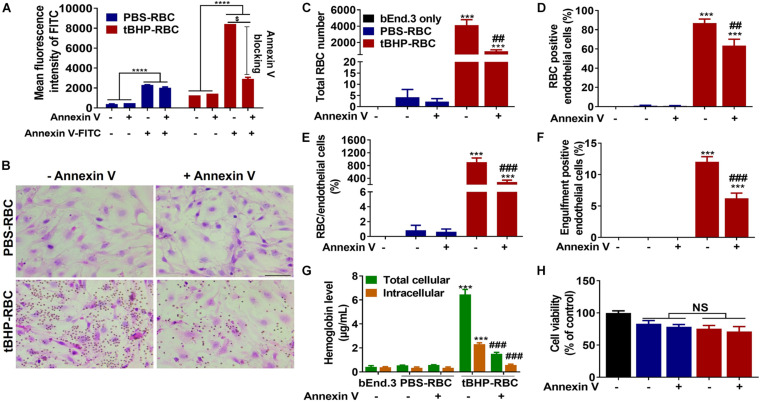
Phosphatidylserine exposure plays an important role in brain endothelial erythrophagocytosis. Phosphatidylserine exposure of tBHP-RBCs was blocked by annexin-V **(A)**. Both RBC adhesion and engulfment were significantly suppressed by annexin-V **(B–F)**. The total cellular and intracellular hemoglobin levels were significantly reduced by annexin-V following a 48-h incubation **(G)**. Erythrophagocytosis did not alter brain endothelial cell viability following the 48-h incubation **(H)**. RBCs are seen as small red/brown dots, and bEnd.3 nuclei are stained purple with hematoxylin in panel **(B)**. Data are presented as mean ± SEM of three independent experiments done in duplicate. One-way **(H)** or two-way **(A,C–G)** ANOVA with Holm–Sidak *post hoc* test was used. ***p < 0.001, ****p < 0.0001 compared with the corresponding bEnd.3-only group and PBS-RBC group; ^##^*p* < 0.01, ^###^*p* < 0.001 compared with tBHP-RBCs without annexin-V; ^$^*p* < 0.0001. Scale bar = 50 μm. NS is “not significant”.

We used annexin-V to mask the externalized phosphatidylserine on the surface of tBHP-RBCs. As shown in Figure 3A, preincubation of tBHP-RBCs with 7.5 μg of annexin-V followed by incubation with annexin-V-FITC reduced the annexin-V-FITC signal equivalent to PBS-control values, indicating complete cloaking of tBHP-induced phosphatidylserine exposure (Figure 3A). Phosphatidylserine masking by annexin-V led to a significant decrease in brain endothelial erythrophagocytosis. As shown in Figure 3, there was a 78% decrease in the adhesion of tBHP-RBCs, a 69% decrease in the number of RBCs attached per bEnd.3 cell, and a 28% decrease in bEnd.3 cells positive for RBCs (Figures 3B–E). Further, the bEnd.3 cells positive for engulfed RBCs were reduced by 48% (Figure 3F). No significant change in RBC adhesion and/or engulfment was observed in the control PBS-RBC group in the presence of annexin-V (Figures 3B–F).

To determine the impact of phosphatidylserine masking on cellular levels of the iron-rich RBC degradation product, hemoglobin, we measured the total and intracellular hemoglobin levels in bEnd.3 cells following a 48-h incubation with RBCs and annexin-V. In the absence of phosphatidylserine cloaking, the total and intracellular hemoglobin levels of bEnd.3 cells incubated with tBHP-RBCs were 6.5 ± 0.4 μg/ml and 2.3 ± 0.1 μg/ml, respectively (Figure 3G). These hemoglobin levels were significantly higher than those in the bEnd.3-only group (without RBCs) and PBS-RBC group (Figure 3G), further supporting the H&E results (Figures 3B–F). In the presence of annexin-V, the total and intracellular hemoglobin levels in bEnd.3 cells incubated with tBHP-RBCs decreased by 77% and 75%, respectively (Figure 3G). No significant difference in bEnd.3 cell viability was observed after incubation with PBS- or tBHP-RBCs for 48 h, and in the presence of annexin-V (Figure 3H).

### Suppression of Brain Endothelial Erythrophagocytosis by Vitamin C

**FIGURE 4:**
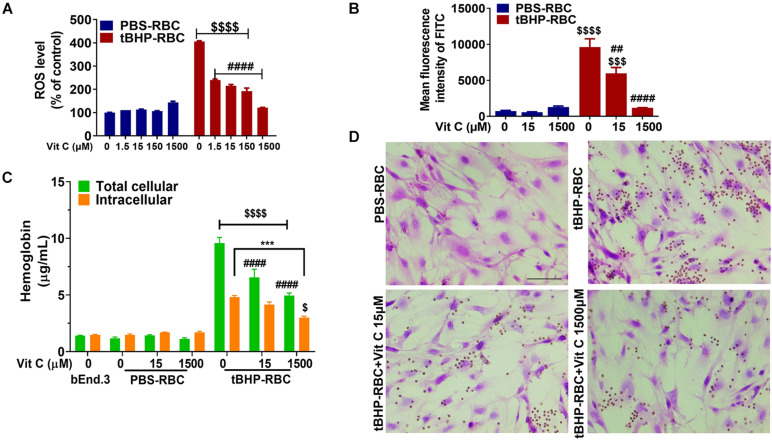
Vitamin C (Vit C) reduced brain endothelial erythrophagocytosis. ROS level **(A)** and phosphatidylserine exposure **(B)** were suppressed by Vit C (15–1,500 μM) after incubation with tBHP-RBCs. The total cellular and intracellular hemoglobin levels of bEnd.3 cells treated with tBHP-RBCs were significantly reduced following a 48-h incubation with Vit C **(C)**. Representative H&E-stained images of bEnd.3 cells following the 48-h incubation with Vit C-treated RBCs **(D)**. RBCs are seen as small red/brown dots, and bEnd.3 nuclei are stained purple with hematoxylin in panel **(D)**. Data are presented as mean ± SEM of three independent experiments done in duplicate. Two-way ANOVA with Holm–Sidak *post hoc* test was used. ***p < 0.001 as indicated. ^##^*p* < 0.01, ^####^*p* < 0.0001 compared with the corresponding tBHP-RBC + Vit C 0 μM group. ^$^*p* < 0.05, ^$$$^*p* < 0.001, ^$$$$^*p* < 0.0001 tBHP-RBC group compared with the corresponding PBS-RBC and bEnd.3-only groups.

Since tBHP is a robust oxidative inducer and causes phosphatidylserine exposure in RBCs, we investigated if suppression of ROS by antioxidant vitamin C alters phosphatidylserine externalization. A 1-h incubation with vitamin C (1.5–1,500 μM) led to a significant reduction of ROS level in tBHP-RBCs (Figure 4A) but had no effect on PBS-RBCs. Notably, vitamin C at a concentration of 1,500 μM led to a full abolishment of ROS accumulation in tBHP-RBCs (Figure 4A). Vitamin C (15 and 1,500 μM) also reduced phosphatidylserine exposure of tBHP-RBCs by 38% and 88%, respectively (Figure 4B). The above prompted us to investigate the effect of free radical scavenging on brain endothelial erythrophagocytosis. As shown in Figure 4C, vitamin C (15 and 1,500 μM) treatment reduced the total cellular hemoglobin level in bEnd.3 cells by 30–47%. Vitamin C (1,500 μM) treatment resulted in a 31% reduction of intracellular hemoglobin level, which shows that vitamin C reduced the engulfment of tBHP-RBCs by the bEnd.3 cells. These results were confirmed by H&E staining (Figure 4D).

### Intracellular Trafficking of RBCs in Brain Endothelial Cells

**FIGURE 5:**
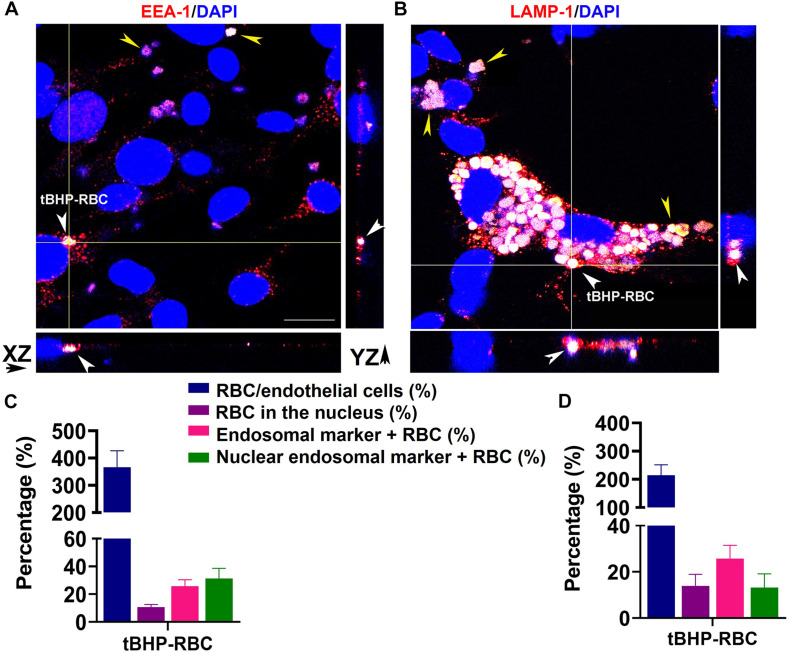
A subset of tBHP-RBCs localized to early and late endosomes. bEnd.3 cells were stained for early endosome antigen 1 (EEA-1; red) **(A)**, lysosome–associated membrane protein 1 (LAMP-1; red) **(B)**, and 4′,6-diamidino-2-phenylindole (DAPI; blue) after a 48-h incubation with PBS- or tBHP-RBCs. RBCs are indicated by yellow arrowheads in panels **(A,B)**. Massive accumulation of tBHP-RBCs within bEnd.3 cells is shown in panel **(B)**. Orthogonal projections from z-stacks of confocal images show tBHP-RBCs co-localized with the endosomal markers in bEnd.3 cells (white arrowheads in **A,B**). Scale bar = 20 μm. Quantification of RBC attachment (expressed as % of number of bEnd.3 cells), RBCs localized to the nucleus (expressed as % of total RBCs; also see **Supplementary Figure 2**), EEA-1- and LAMP-1-positive RBCs (expressed as % of total RBCs), and RBCs in the nucleus positive for EEA-1-/LAMP-1 (expressed as a % of RBCs in the nucleus) (EEA-1: **C** and LAMP-1: **D**). All experiments were repeated at least three times.

We investigated the trafficking of RBCs within the bEnd.3 cells by immunofluorescence to visualize the engulfed RBCs in early and late endosomes. Figures 5A,B show tBHP-RBCs (white/yellow) surrounded by an early endosomal marker, EEA-1 (red), and a late endosomal marker, LAMP-1 (red), within the bEnd.3 cells. 3D reconstructions of the z-stack images confirmed the internalization of tBHP-RBCs in the bEnd.3 cells (Supplementary Figure 2). Our quantification results show that ∼26% of tBHP-RBCs were co-localized with EEA-1- and LAMP-1-positive vesicles (Figures 5C,D). Interestingly, ∼12–15% of tBHP-RBCs were found to co-localize with the nuclear stain DAPI (Figures 5C,D and Supplementary Figure 2).

### Increase in Cellular and Abluminal Iron Following Brain Endothelial Erythrophagocytosis

**FIGURE 6:**
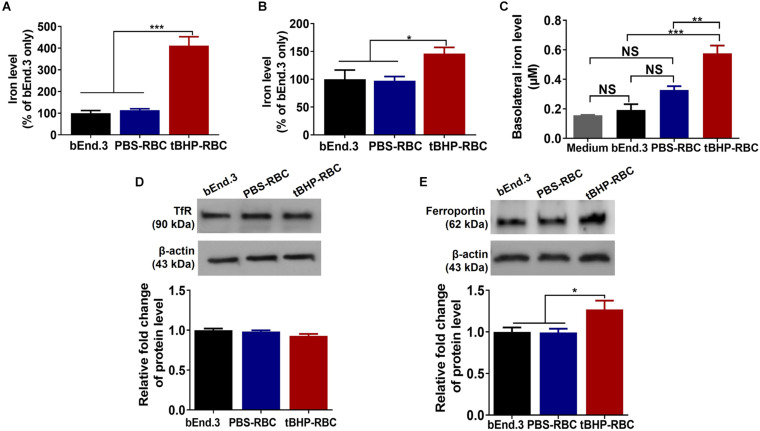
Brain endothelial erythrophagocytosis increased cellular iron load and abluminal iron. Significant increase in total cellular iron (iron from attached and engulfed RBCs) **(A)** and intracellular iron (iron from engulfed RBC) **(B)** in bEnd.3 cells following a 48-h incubation with tBHP-RBCs. Significant increase in basolateral iron levels after the 48-h incubation with tBHP-RBCs **(C)**. No change in the levels of iron importer, transferrin receptor (TfR) **(D)**, but an increase in the levels of iron exporter, ferroportin **(E)**, in bEnd.3 cells after the 48-h incubation with PBS- or tBHP-RBCs. Data are presented as mean ± SEM of three independent experiments done in duplicates. One-way ANOVA with Holm–Sidak *post hoc* test was used. *p < 0.05, **p < 0.01, ***p < 0.001. NS is “not significant”.

For brain endothelial erythrophagocytosis to produce iron-rich lesions in the brain, the former must be accompanied with an increase in intracellular iron and subsequent basolateral (abluminal) iron. We therefore measured the accumulation of iron in the bEnd.3 cells and the basolateral iron across the endothelial monolayer by ICP-MS. After a 48-h incubation with tBHP-RBCs, total cellular iron levels (iron from attached and engulfed RBCs) of bEnd.3 cells increased by 311% compared with bEnd.3-only and 298% compared with PBS-RBC groups (Figure 6A). To further investigate the impact of RBC engulfment on intracellular iron levels, bEnd.3 cells were washed with distilled water to lyse the attached RBCs on the endothelial surface. Intracellular iron levels increased 49% after the incubation of the bEnd.3 cells with tBHP-RBCs compared with PBS-RBCs (Figure 6B). An *in vitro* Transwell^®^ system was used to measure iron transmigration across the bEnd.3 monolayer. At 48 h after RBC incubation, basolateral iron levels in the tBHP-RBC group were 205% and 76% higher than those in the bEnd.3-only group and PBS-RBC group, respectively (Figure 6C). To rule out the contribution of free iron in the apical medium to the increased basolateral iron levels in Figure 6C, we measured free iron levels in the media from PBS- or tBHP-RBCs (in the absence of bEnd.3 cells). No significant differences were observed in the levels of free iron over the course of 48 h between the PBS and tBHP groups (Supplementary Figure 3A). No changes in transendothelial electrical resistance (Supplementary Figures 3B,C) were observed following PBS- and tBHP-RBC incubation. Further, we investigated the protein levels of the major iron influx transporter, the TfR, and the iron exporter, ferroportin. After a 48-h incubation with tBHP-RBCs, no significant change in TfR protein levels was observed compared with the bEnd.3-only group and PBS-RBC group (Figure 6D). A significant 27% increase in ferroportin protein levels was observed in the bEnd.3 cells incubated with tBHP-RBCs compared with the bEnd.3-only and PBS-RBC groups (Figure 6E).

### *In vivo* Prussian Blue-Positive CMH-Like Lesion Development

**FIGURE 7:**
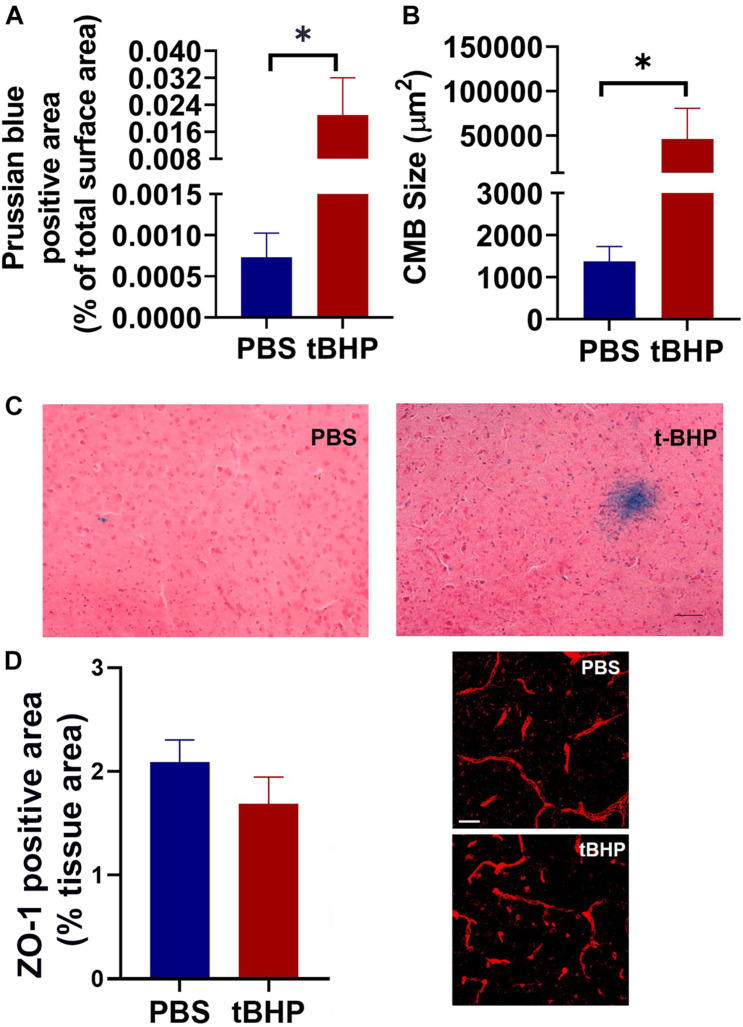
*In vivo* iron-rich Prussian blue-positive cerebral microhemorrhage (CMH)-like lesion development. Aged mice injected with tBHP-RBCs had a higher total Prussian blue-positive lesion area **(A)** and individual lesion size **(B)** compared with the mice injected with PBS-RBCs. Representative Prussian-blue-positive lesions in mice injected with PBS- or tBHP-RBCs **(C)**. No significant difference in the ZO-1-positive area between the mice injected with tBHP-RBCs and PBS-RBCs was observed **(D)**. Data are presented as mean ± SEM of *n* = 3 per group. Scale bar = 200 μm in panel **(C)** and 25 μm in panel **(D)**. ^∗^*p* = 0.05 with a Mann–Whitney U test.

To determine if tBHP-RBCs produce iron-rich CMH-like lesions, aged mice were injected with PBS- or tBHP-RBCs. The mice injected with PBS- or tBHP-RBCs recovered quickly after intravenous RBC injection, appeared normal, and survived the duration of the study (7 days). All aged mice injected with either PBS-RBCs or tBHP-RBCs developed Prussian blue-positive iron-rich lesions. The total Prussian blue-positive area (expressed as a % of brain tissue area analyzed) and size of the individual lesion were, however, greater (*p* = 0.05) in mice injected with tBHP-RBCs than in mice injected with PBS-RBCs (Figures 7A–C). No significant difference was observed in the blood–brain barrier tight-junction protein ZO-1-positive area between the mice injected with PBS-RBCs or tBHP-RBCs (Figure 7D).

## Discussion

Our previous work showed that oxidatively stressed RBCs can undergo robust erythrophagocytosis by the brain endothelium, and this process was accompanied by an increase in the migration of an iron-rich RBC degradation product (hemoglobin) and RBCs across the brain endothelium, *in vitro* and *in vivo*, in the absence of vascular disruption (Chang R. et al., 2018). In the present report, we elucidate the cellular mechanisms that contribute to brain endothelial erythrophagocytosis. We show that phosphatidylserine cloaking and oxidative stress reduction significantly attenuate brain endothelial erythrophagocytosis in the *in vitro* murine brain microvascular endothelial cell model. Further, brain endothelial erythrophagocytosis alters endothelial iron homoeostasis and increases abluminal (brain) iron, without loss of the brain endothelial monolayer integrity, *in vitro*. Finally, we provide histological evidence for increased iron-rich Prussian blue-positive lesion load in mice injected with tBHP-RBCs, correlating with the *in vitro* increase in abluminal iron.

RBCs have a life span of 120 days in humans and about 45 days in mice (Gottlieb et al., 2012). ROS accumulation has been suggested to be a key RBC-life-span determinant by causing phosphatidylserine scrambling and externalization. Phosphatidylserine exposure contributes to the recognition of old/damaged RBCs by altering RBC adhesion to professional phagocytes (macrophages), which is followed by erythrophagocytosis and RBC clearance from the circulation (Franco, 2012; Gottlieb et al., 2012). Besides professional phagocytes, other cell types such as smooth muscle cells can phagocytose phosphatidylserine-exposing apoptotic bodies and aged RBCs (Garfield et al., 1975). Endothelial cells, which are in close contact with circulating RBCs, are also reported to have an erythrophagocytic phenotype. Fens et al. (2010) reported that peripheral endothelial cells can phagocytose RBCs subjected to increased oxidative stress and phosphatidylserine exposure. Similarly, a recent study showed that RBC aging and glycation increases phosphatidylserine externalization and oxidative stress in RBCs, which is associated with enhanced RBC phagocytosis by the vascular endothelium (Catan et al., 2019). Overall, the contribution of the endothelium to erythrophagocytosis and clearance of injured RBCs is now increasingly reported, as is the involvement of phosphatidylserine exposure and oxidative stress in endothelial erythrophagocytosis. Our recent work extended these findings to the brain endothelium and showed robust brain endothelial erythrophagocytosis of phosphatidylserine exposing tBHP-RBCs (Chang R. et al., 2018). To establish the definitive contribution of RBC phosphatidylserine exposure to brain endothelial erythrophagocytosis, herein we cloaked phosphatidylserine with annexin-V which has a high binding affinity for phosphatidylserine in a calcium-dependent manner (Figure 3A; Lizarbe et al., 2013). Cloaking of RBC phosphatidylserine with annexin-V significantly reduced erythrophagocytosis of tBHP-RBCs by the bEnd.3 cells (Figures 3B–F). Annexin-V also significantly reduced total and intracellular hemoglobin, an iron-rich RBC degradation product, in the bEnd.3 cells (Figure 3G), confirming the role of RBC surface phosphatidylserine exposure in brain endothelial erythrophagocytosis. Mechanistically, studies show that phosphatidylserine-dependent erythrophagocytosis by endothelial cells is mediated by the α_v_ integrins or phosphatidylserine-recognizing transmembrane receptors, stabilin-1 and stabilin-2 (Lee et al., 2011; Fens et al., 2012). Besides these, phosphatidylserine-recognizing receptors, including tyrosine kinase receptors, CD300, T-cell immunoglobulin, and mucin domain 1 and 4, are involved in the erythrophagocytosis of phosphatidylserine-exposing RBCs by macrophages, but the involvement of these receptors in endothelial erythrophagocytosis has not been reported and needs further investigation (Klei et al., 2017; Chang C. F. et al., 2018).

Besides RBC phosphatidylserine exposure, tBHP-RBCs showed a dose-dependent increase in intracellular ROS levels in the current study (Figure 1). Given that tBHP is a potent oxidative stressor (Fens et al., 2012; Chang R. et al., 2018), we investigated the involvement of oxidative stress in brain endothelial erythrophagocytosis. For this, we incubated tBHP-RBCs with vitamin C, a ROS scavenger. Vitamin C treatment resulted in a 90% reduction of ROS accumulation and phosphatidylserine exposure of tBHP-RBCs (Figures 4A,B). Accordingly, we observed a significant reduction in erythrophagocytosis of tBHP-RBCs, indicating that both ROS production and phosphatidylserine exposure play a vital role in the brain endothelial erythrophagocytosis (Figure 4). Notably, endothelial cell viability was not altered with brain endothelial erythrophagocytosis up to 48 h, which is consistent with the observations in human peripheral vascular endothelial cells (Catan et al., 2019). It should be noted that despite the almost complete reversal of phosphatidylserine exposure with phosphatidylserine masking with annexin-V (Figure 3) and the highest concentration of vitamin C (Figure 4), brain endothelial erythrophagocytosis was not completely abolished by these agents. These results suggest the involvement of alternate molecular mechanisms of brain endothelial erythrophagocytosis. In this regard, studies show the involvement of vascular cell adhesion molecule 1 (VCAM-1) and intercellular adhesion molecule 4 (ICAM-4) in erythrocyte adhesion to endothelial cells under pathological conditions (Wautier and Wautier, 2013). The expression of these adhesion molecules is increased by pro-inflammatory cytokines, including TNFα. Although we did not study the role of these adhesion molecules on brain endothelial erythrophagocytosis, treatment of brain endothelial cells and tBHP-RBCs with TNFα significantly enhanced brain endothelial erythrophagocytosis in a dose-dependent manner (Supplementary Figure 1), showing the role of inflammation in potentiating brain endothelial erythrophagocytosis, *in vitro*. Besides phosphatidylserine and adhesion molecules, CD47 on the RBC surface is a well-studied “eat me” or “don’t eat me” signal and regulates erythrophagocytosis through its interaction with signal-regulatory protein alpha that is expressed on phagocytes including endothelial cells (Burger et al., 2012). We found a significant 27% reduction in CD47 expression in tBHP-RBCs compared with PBS-RBCs (Supplementary Figure 4). Future work will be needed to determine the role of the CD47-signal-regulatory protein alpha pathway in brain endothelial erythrophagocytosis.

The intracellular processing of RBCs by macrophages following erythrophagocytosis has been extensively studied and involves engulfment of the damaged/injured RBCs into phagosomes *via* phagocytosis, fusion of the phagosome with the lysosome, lysosomal degradation of the RBC, and release of RBC degradation products into the cytosol (Korolnek and Hamza, 2015). Before fusing with the lysosomes, the phagosomes undergo a regulated sequence of fusion events, which involves fusion with the organelles in the endocytic pathway (Duclos et al., 2000). The endocytic pathway is composed of several distinct compartments based on their functions. For example, early endosomes play a primary role in sorting and recycling, while late endosomal compartments, such as lysosomes, serve as a final destination for proteolytic degradation (Hu et al., 2015). Our results demonstrate that a subset of internalized tBHP-RBCs is co-localized with both early and late endosome markers (Figure 5). To examine the fate of the remaining attached RBCs, tBHP- or PBS-RBCs were added to the bEnd.3 monolayer, incubated for 48 h, after which the bEnd.3 media were replaced with fresh media, and the attached RBCs were incubated with the bEnd.3 cells for another 72 h (all the free-floating RBCs were removed with the medium change). The tBHP-RBCs attached to the bEnd.3 cells after 72 h (RBCs appear as black spots in Supplementary Figure 2D) were reduced. The total cellular iron levels in the tBHP-RBC-treated bEnd.3 cells were not significantly different from the PBS-RBC-treated bEnd.3 cells, suggesting that the tBHP-RBCs were not present inside the bEnd.3 cells (data not shown). When we measured the iron in the media to determine if the RBCs had internalized and degraded and the resulting iron released to the media, we found a small but significantly higher iron level in the media collected from the bEnd.3 cells incubated with tBHP-RBCs compared with PBS-RBCs (Supplementary Figure 2E). These results suggest that with time, the engulfed RBCs are degraded within the lysosome, as implicated by RBCs co-localized with LAMP-1 (Figure 5), and the resulting iron released into the media. Contrary to this, a recent study showed that microspheres and emboli are retained in the brain endothelial cells without degradation, triggering their transmigration (van der Wijk et al., 2020). Our previous *in vivo* work showed a similar phenomenon of RBC migration across the brain endothelium (Chang R. et al., 2018), and some of the engulfed RBCs may eventually transmigrate across the brain endothelium. Overall, although the massive engulfment of tBHP-RBCs by some endothelial cells may overwhelm the abovementioned clearance mechanisms at some point, increase the intracellular iron pool, and trigger endothelial cell apoptosis, we did not observe overt endothelial cell death up to 5 days (48 + 72 h) following RBC incubation (Supplementary Figure 2D). However, we, cannot rule out apoptosis, although only a fraction of HUVEC cells showed signs of apoptosis post-erythrophagocytosis following 24 h of incubation (Fens et al., 2012).

We observed RBCs not only in the cytosol but also in the nucleus (Figure 5 and Supplementary Figure 2). Trafficking of cargo to the nucleus has been reported previously and been shown to impact cellular function and regulate gene expression (Olsnes et al., 2003; Chaumet et al., 2015). Further, cellular trafficking is dependent on the size of cargo, such that large cargos (3 μm) are directed to the nucleus for digestion (Keller et al., 2017). It is therefore conceivable that the large size of the RBC sorts them to the nucleus in the current study. This finding of RBC trafficking to the endothelial cell nucleus is novel, and its implication needs to be further investigated.

In the current study, brain endothelial erythrophagocytosis was accompanied with a significant increase in the intracellular iron pool (Figure 6), which is consistent with previous work showing an increase in cellular iron load with erythrophagocytosis by professional phagocytes (Knutson et al., 2003). Additionally, and more relevant from the standpoint of iron-rich CMH-like lesion development, we showed an increase in migration of iron across an intact endothelial cell monolayer, providing direct evidence for increased abluminal iron with brain endothelial erythrophagocytosis. These results build on our prior report that brain endothelial erythrophagocytosis was accompanied by passage of hemoglobin across the brain endothelial monolayer with unaltered brain endothelial monolayer integrity (Chang R. et al., 2018). Sources of abluminal iron in the current study could be hemoglobin or cytosolic iron (iron released after lysosomal degradation within the brain endothelial cells) which migrates across the brain endothelial monolayer. These results show that brain endothelial erythrophagocytosis is associated with an increase in abluminal (brain facing) iron load. Once in the brain parenchyma, microglial cells are well equipped to clear RBC and its degradation products, including hemoglobin, *via* scavenger receptors including CD36, CD47, CD163, and CD91 (Pan et al., 2020). Within the microglial cells, hemoglobin can be broken down into free heme followed by free iron that is stored as the insoluble hemosiderin-iron. Similarly, brain iron homeostasis is tightly regulated by the blood–brain barrier transferrin receptors, apotransferrin released by the oligodendrocytes, and iron transporters present on the ependymal surface. Transferrin-bound iron can also be taken up by astrocytes or neurons that express transferrin receptors. Any excess iron released from hemoglobin degradation may be stored as ferritin-iron (Wagner et al., 2003).

Increased cellular iron load after erythrophagocytosis is associated with alterations in iron transport proteins (Knutson et al., 2003). To elucidate the impact of brain endothelial erythrophagocytosis on the brain endothelial iron transporters, we measured levels of TfR, the major iron influx transporter, and ferroportin, the only known iron exporter at the blood–brain barrier (Ganz, 2005; Moos et al., 2007; McCarthy and Kosman, 2015). TfRs are localized to the luminal and abluminal surface of the brain microvasculature (Huwyler and Pardridge, 1998) and facilitate iron uptake into the brain microvascular endothelial cells and export into the brain *via* receptor-mediated transcytosis (Moos et al., 2007). Similarly, studies show that ferroportin is also localized to both the luminal and abluminal surfaces of the brain microvasculature to facilitate iron export from the brain endothelial cells (Wu et al., 2004; McCarthy and Kosman, 2015). In the current study, brain endothelial erythrophagocytosis was not associated with a change in TfR protein levels; however, ferroportin protein levels were significantly increased (Figure 6). This is consistent with an increase in ferroportin levels following erythrophagocytosis by macrophages (Knutson et al., 2003) and may be a compensatory response to maintain iron homeostasis and prevent iron overloading within the endothelial cells (Chiou et al., 2019). This also provides a potential explanation for why the brain endothelial cell viability and brain endothelial integrity are not compromised despite robust erythrophagocytosis and cellular iron increase (Figure 3 and Supplementary Figure 3).

Our previous work provided *in vivo* evidence for migration of tBHP-RBCs across an intact brain endothelium in Tie2-GFP mice, while the PBS-RBCs remained confined to the vasculature (Chang R. et al., 2018). These findings were recently corroborated by a study showing brain endothelial erythrophagocytosis of Plasmodium falciparum-infected RBCs *in vivo* (Adams et al., 2021). In the current study, we show that mice injected with tBHP-RBCs had a greater Prussian blue-positive CMH-like lesion load compared with mice injected with PBS-RBCs (Figure 7), supporting the *in vitro* findings of increased abluminal iron with brain endothelial erythrophagocytosis (Figure 6). Our previous work using *ex vivo* microscopy demonstrated an intact endothelium at the site of RBC extravasation (Chang R. et al., 2018) as opposed to a ruptured endothelium, which is seen with Prussian blue-positive lesions associated with blood–brain barrier damage (Sumbria et al., 2016). Further, we found no change in the blood–brain barrier tight junction protein ZO-1 seven days after tBHP-RBC injection (Figure 7). This is in line with our recent work showing no association between blood–brain barrier changes with CMH development in humans (Wadi et al., 2020). Accordingly, our *in vitro* data and other reports (Catan et al., 2019) show no change in brain endothelial cell viability and permeability with erythrophagocytosis (Figure 3 and Supplementary Figure 3). However, it is important to note that erythrophagocytosis of glycated RBCs is associated with alterations in endothelial function (e.g., reduction in migration and proliferation), and a small increase in apoptotic markers has been reported with endothelial erythrophagocytosis *in vitro* (Fens et al., 2012; Catan et al., 2019). Additionally, engulfment of microspheres by the brain endothelium and subsequent migration into the brain parenchyma is associated with temporary blood–brain barrier opening in a microembolization rat model (van der Wijk et al., 2020). Therefore, the increased RBC or iron load within the brain endothelial cells might trigger temporal endothelial remodeling or dysfunction resulting in blood–brain barrier changes. These changes may further increase the extravasation of RBCs and/or RBC degradation products into the brain, increasing the likelihood of iron-rich lesion development. Future work using intravital imaging will allow us to examine the causal and temporal relationship between brain endothelial erythrophagocytosis and blood–brain barrier permeability at the site of RBC extravasation using tracers followed by Prussian-blue staining to detect iron-rich lesions. These studies will also help elucidate how widespread brain endothelial erythrophagocytosis is, and if brain endothelial erythrophagocytosis-mediated abluminal iron increase is a focal or a diffuse event. Notably, our *in vivo* study showed focal iron deposits associated with tBHP-RBCs.

**FIGURE 8:**
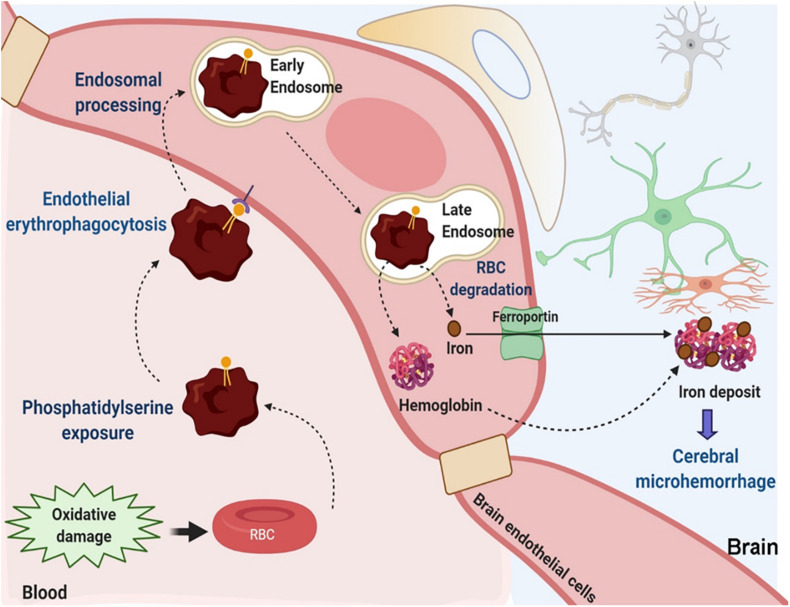
Suggested mechanisms contributing to brain endothelial erythrophagocytosis mediated Prussian blue-positive cerebral lesions. Oxidative stress and phosphatidylserine exposure increase phagocytosis of RBCs by the brain endothelium. Other mechanisms including CD47 reduction may also be involved. Engulfed RBCs undergo endosomal sorting and ultimately undergo degradation within the late endosomes/lysosomes. This increases intracellular pools of iron-rich RBC degradation products, hemoglobin and iron. Free iron may be effluxed from the brain endothelial cells *via* the abluminal iron-exporter ferroportin. Alternatively, hemoglobin may migrate across the brain endothelium. Migration of iron-rich RBC degradation products across the brain endothelial cells can increase the brain abluminal iron, resulting in Prussian blue-positive CMH-like lesion development.

Overall, based on the results of the current study we propose the following sequence of events whereby brain endothelial erythrophagocytosis may result in iron-rich CMH-like lesion development (Figure 8): oxidative stress and phosphatidylserine exposure increase phagocytosis of RBCs by the brain endothelium. Other mechanisms including CD47 reduction may also contribute to this process. A subset of engulfed RBCs undergoes endosomal sorting and degradation. RBC engulfment and degradation increase intracellular pools of iron-rich RBC degradation products, hemoglobin and iron. Free iron may be effluxed from the brain endothelial cells *via* the iron exporter ferroportin. Additionally, hemoglobin may migrate across the brain endothelium (Chang R. et al., 2018). Both iron and hemoglobin migration across the brain endothelial cells increases brain abluminal iron, which results in Prussian blue-positive CMH-like lesion development.

## Data Availability Statement

The original contributions presented in the study are included in the article/Supplementary Material, further inquiries can be directed to the corresponding author.

## Ethics Statement

The animal study was reviewed and approved by University of California-Irvine’s Institutional Animal Care and Use Committee, and carried out in compliance with University Laboratory Animal Resources regulations.

## Author Contributions

RKS and MJF conceptualized the study. JS, PV, RKS, and DHC assisted with the experimental design. JS, PV, SM, and RKS performed the experiments and data analysis. AP-H assisted with the data analysis. ACFN and WLL assisted with the *in vivo* study. JS and RKS wrote the first draft of the manuscript. RKS, DHC, and MJF acquired the funding. All authors discussed the results and their implications, and commented on the manuscript. All authors read and approved the final manuscript.

## Conflict of Interest

The authors declare that the research was conducted in the absence of any commercial or financial relationships that could be construed as a potential conflict of interest.

## Publisher’s Note

All claims expressed in this article are solely those of the authors and do not necessarily represent those of their affiliated organizations, or those of the publisher, the editors and the reviewers. Any product that may be evaluated in this article, or claim that may be made by its manufacturer, is not guaranteed or endorsed by the publisher.

## Abbreviations

CMBs: cerebral microbleeds
CMHs: cerebral microhemorrhages
H_2_DCFDA: 2′,7′-dichlorodihydrofluorescein diacetate
EEA-1: early endosome antigen 1
H&E: hematoxylin and eosin
ICP-MS: inductively coupled plasma mass spectrometry
LAMP-1: lysosome-associated membrane protein 1
PBS: phosphate-buffered saline
RBC: red blood cell
ROS: reactive oxygen species
tBHP: tert-butyl hydroperoxide
TfR: transferrin receptor
ZO-1: zonula occludens - 1.
