# Supplementary material for: Insights Into the Mechanisms of Brain Endothelial Erythrophagocytosis
Source: Front Cell Dev Biol. 2021 Aug 2;9:672009. doi: 10.3389/fcell.2021.672009 (PMC8365766; doi:10.3389/fcell.2021.672009)
Supplement: Supplementary file 1 [file Data_Sheet_1.docx]

**SUPPLEMENTAL MATERIAL**

**Supplemental Figures**


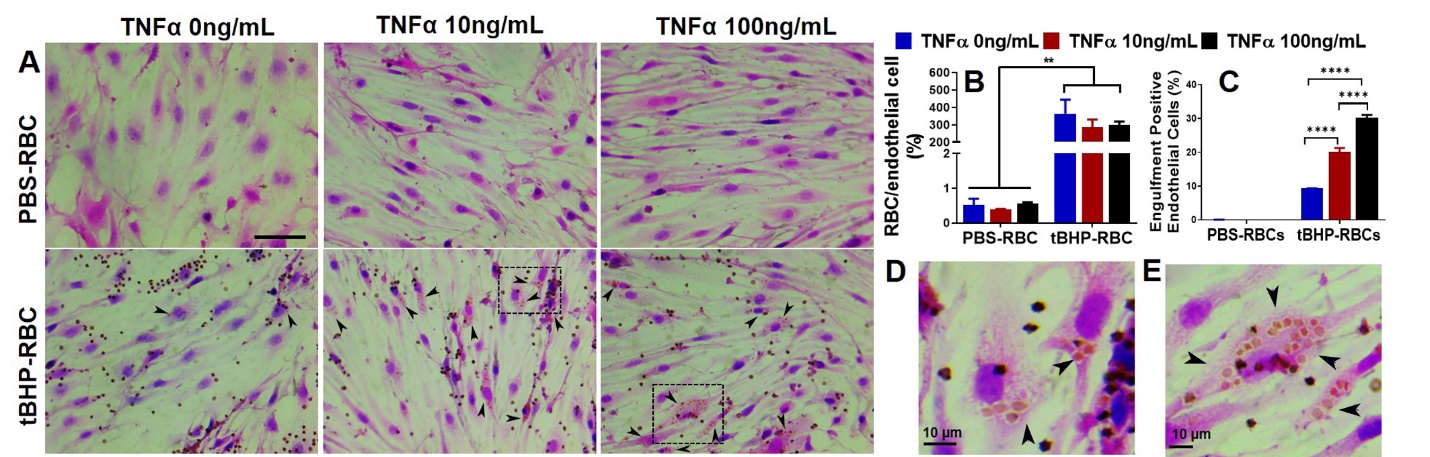


**Supplemental Figure 1. The effect of TNFα on brain endothelial erythrophagocytosis.** bEnd.3 cells were co-incubated with TNFα (0, 10 and 100 ng/mL) and PBS-RBCs or tBHP-RBCs for 48-h and brain endothelial erythrophagocytosis was evaluated using H&E staining and light microscopy. No significant change in RBC adhesion (**A-C**) was observed with TNFα treatment. A dose-dependent increase in tBHP-RBC-engulfment was induced by TNFα (10 and 100 ng/mL) (**C-E**). Black arrowheads indicate bEnd.3 cells with engulfed RBCs. Boxed areas in A show engulfed RBCs with 10 ng/mL and 100 ng/mL TNFα in D and E, respectively. Data are presented as mean ± SEM of 3 independent experiments done in duplicates. Two-way ANOVA with Holm-Sidak post-hoc test was used. **p<0.01, ****p<0.0001. Scale bar = 50 µm in A and 10 µm in D and E.


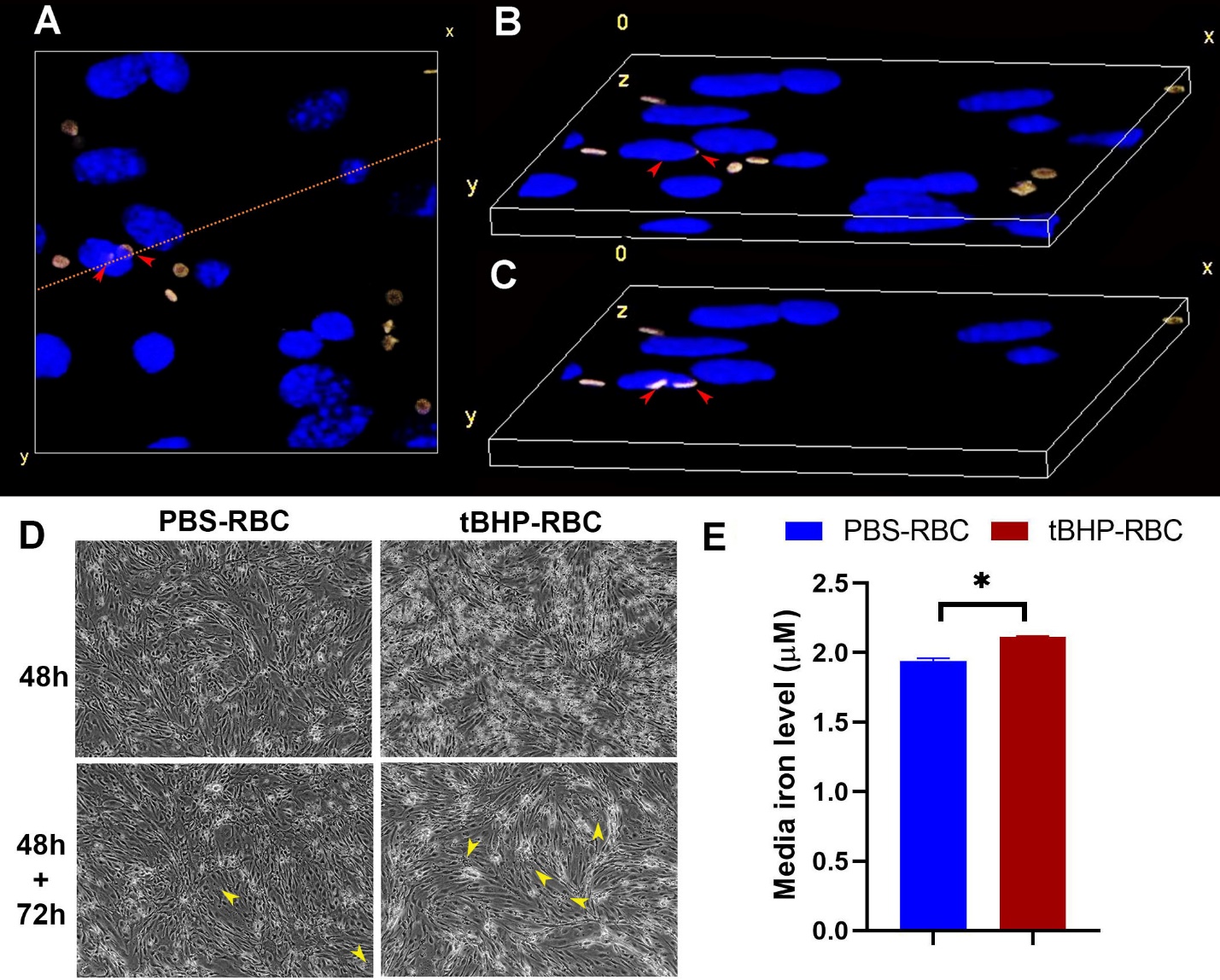


**Supplemental Figure 2. Internalization and localization of tBHP-RBCs within the bEnd.3 nucleus, and the fate of RBCs after prolonged incubation.** XY top view (**A**) and the 3D volume-view of z-stack images (**B**). The cross-sectional volume view (**C**) shows engulfed tBHP-RBCs within the bEnd.3 nucleus. The cross section in C corresponds to the orange line in A. Red arrow heads show the position of the same engulfed RBC in A, B and C. Engulfed RBCs are not seen in the volume-view (**B**) but are seen when the volume-view in cut through (**C**). Autofluorescence of tBHP-RBCs is yellow and nucleus is stained with DAPI (blue). Incubation of PBS- or tBHP-RBCs with bEnd.3 cells for 48-h followed by replacement with fresh media and incubation for an additional 72-h (48-h+72-h) decreased tBHP-RBCs attachment to the bEnd.3 cells. RBCs appear as small black dots as shown by the yellow arrowheads (**D**). There was a small significant increase in the iron in the media of bEnd.3 cells incubated with tBHP-RBCs for 48-h+72-h (**E**). Data are presented as mean ± SEM of 3 independent experiments. Student’s *t-*test was used in E. *p<0.05.


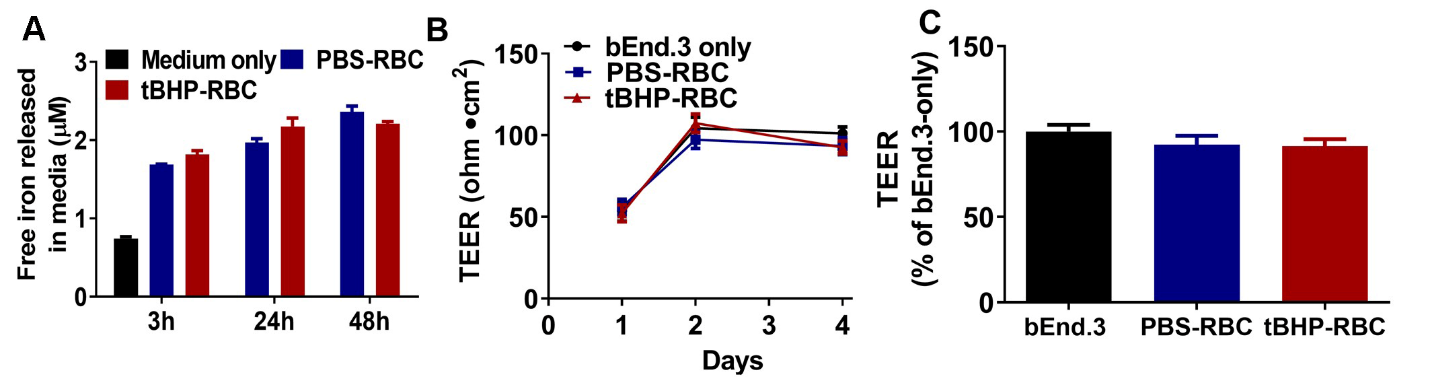
**Supplemental Figure 3.** PBS- or tBHP-treated RBCs were incubated in media over 48-h (no bEnd.3 cells were used for this assay) and media samples were collected at indicated times. Media was filtered to separate RBCs, and free iron in the media was measured using ICP-MS. No significant change in free iron levels was observed between the PBS- and tBHP-treated RBCs (**A**). The integrity of bEnd.3 monolayer in transwells of 6 well plate (pore size 0.4 µm) was assessed by measuring transendothelial electrical resistance. After seeding cells, transendothelial electrical resistance was measured on day 1 and 2 to allow for stabilization. On day 2, treated RBCs were incubated with bEnd.3 for 48-h and transendothelial electrical resistance was measured on day 4. Transendothelial electrical resistance measurements are presented as ohm · cm^2^ (**B**) and % of bEnd.3-only values on day 4 (**C**). Data are presented as mean ± SEM of 3 independent experiments done in duplicates. Two/one-way ANOVA with Holm-Sidak post-hoc test was used.


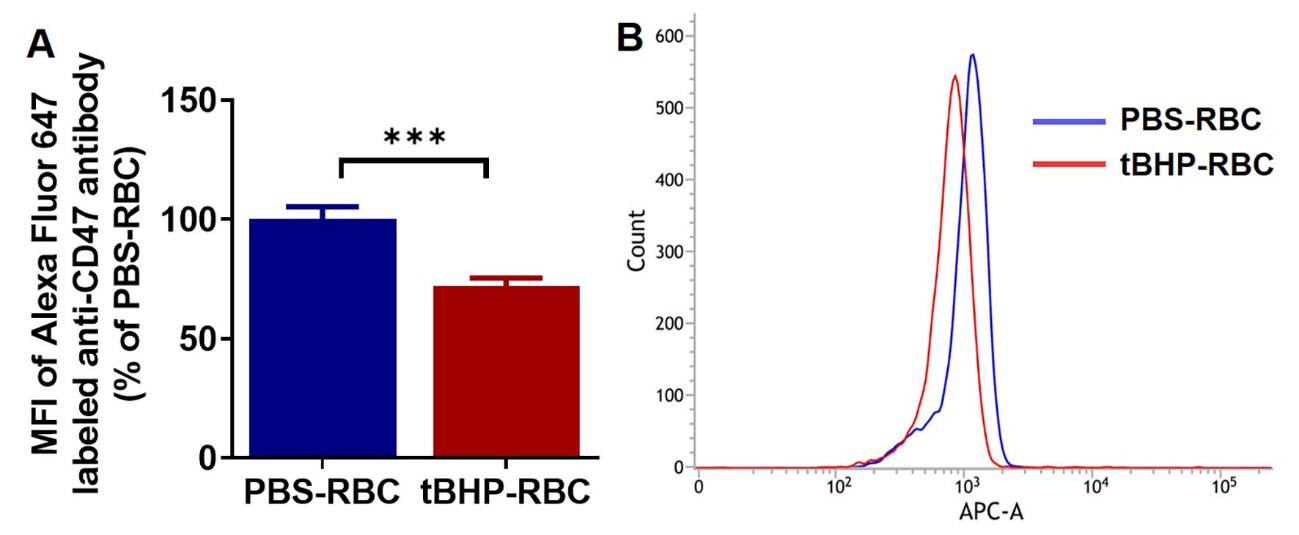


**Supplemental Figure 4. CD47 expression in PBS- and tBHP-RBCs.** CD47 expression on RBCs was measured by flow cytometry after 30 min incubation with PBS or tBHP (**A, B**). Data are presented as mean ± SEM of 4 independent experiments. ***p<0.001 with Student's t-test. MFI: mean fluorescent intensity.

**Major Resources Table**

**Animals (*in vivo* studies)**

| **Species** | **Vendor or Source** | **Background Strain** | **Sex** | **Persistent ID / URL** |
| --- | --- | --- | --- | --- |
| Mice | National Institute of Aging | C57BL6 | Male |  |

**Antibodies**

| **Target antigen** | **Vendor or Source** | **Catalog #** | **Working concentration** | **Persistent ID / URL** |
| --- | --- | --- | --- | --- |
| EEA-1 | Santa Cruz Biotechnology | sc-365652 AF647 | 1:50 | <https://www.scbt.com/p/eea1-antibody-e-8> |
| LAMP-1 | Developmental Studies Hybridoma Bank | 1D4B-s | 1:10 | <https://dshb.biology.uiowa.edu/1D4B> |
| β-Actin | Santa Cruz Biotechnology | sc-47778 | 1:100 | https://www.scbt.com/p/beta-actin-antibody-c4 |
| Alexa Fluor 647 Goat anti-rat IgG | Thermofisher | A-21247 | 1:200 | <https://www.thermofisher.com/antibody/product/Goat-anti-Rat-IgG-H-L-Cross-Adsorbed-Secondary-Antibody-Polyclonal/A-21247> |
| Transferrin receptor | Thermofisher | 13-6800 | 1:1000 | https://www.thermofisher.com/antibody/product/Transferrin-Receptor-Antibody-clone-H68-4-Monoclonal/13-6800 |
| Ferroportin | Novus Biologicals, LLC | NBP1-21502SS | 1:1000 | <https://www.novusbio.com/products/ferroportin-slc40a1-antibody_nbp1-21502> |
| Anti-mouse IgG Secondary, HRP conjugated (secondary for anti-transferrin receptor antibody) | Thermofisher | G-21040 | 1:10000 | https://www.thermofisher.com/antibody/product/Goat-anti-Mouse-IgG-H-L-Cross-Adsorbed-Secondary-Antibody-Polyclonal/G-21040 |
| mouse-IgGκbinding protein-HRP conjugated (secondary for anti-β-actin antibody) | Santa Cruz Biotechnology | sc-516102 | 1:1000 | https://www.scbt.com/p/m-igg-kappa-bp-hrp |
| Anti-rabbit IgG, HRP-linked Antibody (secondary for anti-ferroportin antibody) | Cell Signaling Technology | CST-7074 | 1:1000 | https://www.cellsignal.com/products/secondary-antibodies/anti-rabbit-igg-hrp-linked-antibody/7074 |
| Anti-CD47 | Santa Cruz Biotechnology | sc-12731 | 1:200 | https://www.scbt.com/p/cd47-antibody-miap301 |
| ZO-1 Polyclonal Antibody | Invitrogen | 40-2200 | 1:100 | https://www.thermofisher.com/antibody/product/ZO-1-Antibody-Polyclonal/40-2200 |
| Goat anti-Rabbit IgG (H+L) | Invitrogen | A27040 | 1:400 | https://www.thermofisher.com/antibody/product/Goat-anti-Rabbit-IgG-H-L-Secondary-Antibody-Recombinant-Polyclonal/A27040 |

**Cultured Cells**

| **Name** | **Vendor or Source** | **Sex (F, M, or unknown)** | **Persistent ID / URL** |
| --- | --- | --- | --- |
| bEnd.3 | ATCC | unknown | https://www.atcc.org/Products/All/CRL-2299.aspx#documentation |

**Other**

| **Description** | **Source / Repository** | **Persistent ID / URL** |
| --- | --- | --- |
| Annexin V-FITC | BioLegend Cat No. 640905 | <https://www.biolegend.com/en-us/products/fitc-annexin-v-5161?GroupID=GROUP26> |
| Mouse hemoglobin | Lee Biosolutions Cat No.: 324-30 | <https://www.leebio.com/product/360/hemoglobin-mouse-red-blood-cells-324-30> |
| BALB/C Mouse Red Blood Cells Male | Bioivt | https://bioivt.com/mouse-red-blood-cells |
| Cell Counting Kit-8 | Dojindo Molecular Technologies Cat No. SKU: CK04 | https://www.dojindo.com/product/cell-counting-kit-8/ |
| Purified Annexin V | Biolegend Cat No. 640901 | https://www.biolegend.com/en-us/products/purified-annexin-v-8677 |
| Annexin V Binding Buffer | Biolegend Cat No. 422201 | https://www.biolegend.com/en-us/products/annexin-v-binding-buffer-5162 |
| Tert-butyl hydroperoxide | Sigma Cat No. 458139 | https://www.sigmaaldrich.com/catalog/product/aldrich/458139?lang=en&region=US |
| H2DCFDA | Thermofisher Cat No. D399 | https://www.thermofisher.com/order/catalog/product/D399#/D399 |
| Eosin Y | Sigma Aldrich, Cat No.HT110216 | https://www.sigmaaldrich.com/catalog/product/sigma/ht110216?lang=en&region=US |
| Hematoxylin Solution, Mayer’s | Sigma Aldrich, Cat No. MHS32 | https://www.sigmaaldrich.com/catalog/product/sigma/mhs32?lang=en&region=US |
| Permount | Fisher Scientific Cat No. SP15-100 | https://www.fishersci.com/shop/products/fisher-chemical-permount-mounting-medium-100ml/sp15100 |
| 2,7-diaminofluorene | Sigma Aldrich, Cat No. D17106 | https://www.sigmaaldrich.com/catalog/product/aldrich/d17106?lang=en&region=US |
| Iron (III) nitrate, ferric nitrate (ICP grade) | Agilent Cat No. ICP-326 | https://www.agilent.com/store/en_US/Prod-ICP-326/ICP-326 |
